# Complete Genome Sequences of Mycobacterium smegmatis Phages MelsMeow, Yorick, Virgeve, and Mikro

**DOI:** 10.1128/mra.00758-22

**Published:** 2022-09-26

**Authors:** Victoria J. Frost, Jada E. Fogle, Ryan N. Harris, Brooke Jewell, Kaylee E. Mills, Jessica E. Morgan, Precious T. Thompson, Emi Umemoto, Kristi M. Westover

**Affiliations:** a Department of Biology, Winthrop University, Rock Hill, South Carolina, USA; Queens College CUNY

## Abstract

Mycobacterium phages Mikro, Yorick, Virgeve, and MelsMeow were isolated from soil in Rock Hill, South Carolina. Mikro is a myovirus with a comparatively large genome of 157,166 bp. The remainder are siphoviruses with genome lengths ranging from 59,227 bp to 68,563 bp. All phages were isolated on Mycobacterium smegmatis.

## ANNOUNCEMENT

**FIG 1:**
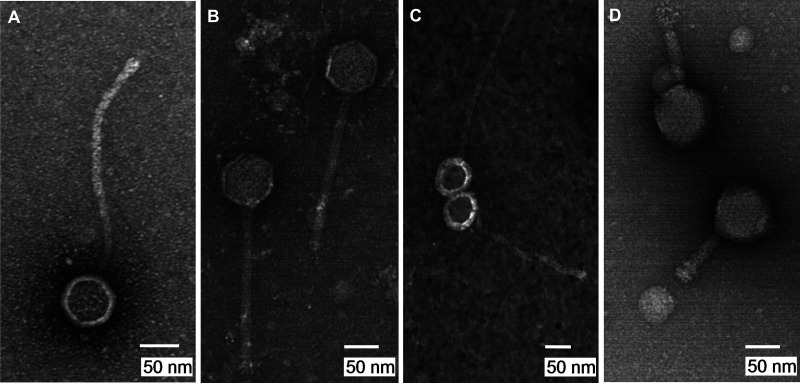
Transmission electron micrographs of Mycobacterium phages MelsMeow (A), Yorick (B), Virgeve (C), and Mikro (D). Phage lysates were negatively stained with 1% uranyl acetate.

Researchers have documented the use of phage therapy to treat multidrug-resistant Mycobacterium infections, with promising results (1, 2). Here, Mycobacterium smegmatis mc^2^155 was used to isolate mycobacteriophages from soil at Winthrop University, Rock Hill, SC. Mycobacteriophages Mikro, Yorick, and Virgeve were isolated from damp soil in a shaded flower bed, whereas MelsMeow was isolated from dry soil close to a tree root (see Table 1 for location coordinates [GPS]), using standard procedures (https://seaphagesphagediscoveryguide.helpdocsonline.com/home). Soil samples were washed for 2 h using 7H9 broth containing 1 mM CaCl_2_ and centrifuged at 4,000 rpm for 10 min, and the supernatant filtered (0.22 μm). A fraction of each filtrate was inoculated with M. smegmatis and shaken (250 rpm) at 37°C for 2 to 4 days to enrich for mycobacteriophages before being refiltered. Both enriched and unenriched filtrates were examined for phage by plating in soft agar with M. smegmatis mc^2^ 155 on 7H9 agar plates and incubating at 37°C. Mikro was isolated from unenriched filtrate and produced very small (<1 mm diameter) clear plaques. Yorick, Virgeve, and MelsMeow were isolated from enriched filtrates. Virgeve and MelsMeow produced clear plaques, while Yorick produced turbid plaques. Transmission electron microscopy revealed that Mikro has a *Myoviridae* morphotype with a short contractile tail. Phages Yorick, Virgeve, and MelsMeow have *Siphoviridae* morphologies with long flexible tails (Fig. 1).

**TABLE 1:** Phage GenBank and SRA accession numbers and genome assembly results

Phage name	Location site (GPS)	Avg coverage (X)	Reads (K)	Cluster	Genome size (bp)	Genome ends	GC content (%)	No. of genes
Mikro	34.940074 N, 81.033099 W	1,503	1,661	C1	157,166	Circular permuted	64.8	260
Yorick	34.940113 N, 81.032921 W	933	385	F1	59,227	3’ single-stranded overhang (5′-CGGTAGGCGC-3′)	61.3	99
Virgeve	34.940113 N, 81.032921 W	630	658.8	B1	68.046	Circular permuted	66.5	99
MelsMeow	34.93712 N, 81.03197 W	646	620.8	B1	68,563	Circular permuted	66.4	100

Phage DNA was extracted using the Wizard DNA cleanup kit (Promega), and libraries were constructed using the NEBNext Ultra II FS DNA library prep kit before sequencing with the Illumina MiSeq v3 platform. Results of the 150-bp single-end raw reads were assembled using Newbler v2.9 and checked for accuracy, coverage, and genomic termini using Consed v29 as previously described (3, 4). Sequencing results and phage genome characteristics are listed in Table 1 and include genome size, GC content, predicted number of genes, and phage cluster designation based on gene content similarity (GCS) of at least 35% to phages within the Actinobacteriophage database (https://phagesdb.org/) using the GCS tool at phagesDB and previously described criteria (5, 6).

Default parameters were used for all bioinformatics analyses. Genome sequences were annotated using DNA Master v5.23.6 (7) embedded with Glimmer v3.02 (8) and GeneMark v2.5 (9), Starterator (7), Phamerator v3 (10), HHpred v2.07 (11), and BLASTp v2.13.0 (12). Transfer RNAs were identified using Aragorn v1.1 integrated in DNA Master (7), Aragorn v1.2.38 (13), and tRNAscan-SE v2.0.6 (14).

Annotation revealed putative gene functions for each phage. MelsMeow and Virgeve genomes begin with rightward-transcribed genes that include a portal protein, capsid maturation protease, MuF-like fusion protein, two-tail assembly chaperones, and a tape-measure protein. The second portion of these genomes include DNA helicase, DNA Pol I, and HNH endonuclease genes that are transcribed leftwards. Neither genome contains identifiable integrases or immunity repressors, and are predicted to be lytic phages. Yorick’s genome, composed of mostly rightward-transcribed genes, includes an immunity repressor, tyrosine integrase, holin, Cro, and an antirepressor, and is therefore predicted to be temperate, consistent with the turbid plaques it produces. Mikro also contains mostly rightward-transcribed genes, as well as many (32) tRNAs.

### Nucleotide sequence accession numbers.

The complete genome sequences of phages Mikro, Yorick, Virgeve, and MelsMeow are available in GenBank (accession numbers ON456344, ON456356, ON456332, and ON456330, respectively). The raw sequencing reads are available in the NCBI SRA under accession numbers SRX15940724, SRX15940726, SRX14485102, and SRX14483218, respectively. The Actinobacteriophage sequencing BioProject accession number is PRJNA488469.

## References

[1] Dedrick RM, Smith BE, Cristinziano M, Freeman KG, Jacobs-Sera D, Belessis Y, Whitney Brown A, Cohen KA, Davidson RM, van Duin D, Gainey A, Garcia CB, Robert George CR, Haidar G, Ip W, Iredell J, Khatami A, Little JS, Malmivaara K, McMullan BJ, Michalik DE, Moscatelli A, Nick JA, Tupayachi Ortiz MG, Polenakovik HM, Robinson PD, Skurnik M, Solomon DA, Soothill J, Spencer H, Wark P, Worth A, Schooley RT, Benson CA, Hatfull GF. 2022. Phage therapy of *Mycobacterium* infections: compassionate-use of phages in twenty patients with drug-resistant mycobacterial disease. Clinical Infectious Diseases ciac453. doi:10.1093/cid/ciac453. 35676823 PMC9825826

[2] Nick JA, Dedrick RM, Gray AL, Vladar EK, Smith BE, Freeman KG, Malcolm KC, Epperson LE, Hasan NA, Hendrix J, Callahan K, Walton K, Vestal B, Wheeler E, Rysavy NM, Poch K, Caceres S, Lovell VK, Hisert KB, de Moura VC, Chatterjee D, De P, Weakly N, Martiniano SL, Lynch DA, Daley CL, Strong M, Jia F, Hatfull GF, Davidson RM. 2022. Host and pathogen response to bacteriophage engineered against *Mycobacterium* abscessus lung infection. Cell 185:1860–1874.e12. doi:10.1016/j.cell.2022.04.024. 35568033 PMC9840467

[3] Gordon D, Green P. 2013. Consed: a graphical editor for next-generation sequencing. Bioinformatics 29:2936–2937. doi:10.1093/bioinformatics/btt515. 23995391 PMC3810858

[4] Miller JR, Koren S, Sutton G. 2010. Assembly algorithms for next-generation sequencing data. Genomics 95:315–327. doi:10.1016/j.ygeno.2010.03.001. 20211242 PMC2874646

[5] Russell DA, Hatfull GF. 2017. PhagesDB: the actinobacteriophage database. Bioinformatics 33:784–786. doi:10.1093/bioinformatics/btw711. 28365761 PMC5860397

[6] Pope WH, Mavrich TN, Garlena RA, Guerrero-Bustamante CA, Jacobs-Sera D, Montgomery MT, Russell DA, Warner MH, Hatfull GF, Science Education Alliance-Phage Hunters Advancing Genomics and Evolutionary Science (SEA-PHAGES). 2017. Bacteriophages of *Gordonia* spp. display a spectrum of diversity and genetic relationships. mBio 8:e01069-17. doi:10.1128/mBio.01069-17. 28811342 PMC5559632

[7] Pope WH, Jacobs-Sera D. 2018. Annotation of bacteriophage genome sequences using DNA Master: an overview. Methods Mol Biol 1681:217–229. doi:10.1007/978-1-4939-7343-9_16. 29134598

[8] Delcher AL, Bratke KA, Powers EC, Salzberg SL. 2007. Identifying bacterial genes and endosymbiont DNA with Glimmer. Bioinformatics 23:673–679. doi:10.1093/bioinformatics/btm009. 17237039 PMC2387122

[9] Besemer J, Borodovsky M. 2005. GeneMark: web software for gene finding in prokaryotes, eukaryotes and viruses. Nucleic Acids Res 33:W451–W454. doi:10.1093/nar/gki487. 15980510 PMC1160247

[10] Cresawn SG, Bogel M, Day N, Jacobs-Sera D, Hendrix RW, Hatfull GF. 2011. Phamerator: a bioinformatic tool for comparative bacteriophage genomics. BMC Bioinformatics 12:395. doi:10.1186/1471-2105-12-395. 21991981 PMC3233612

[11] Söding J, Biegert A, Lupas AN. 2005. The HHpred interactive server for protein homology detection and structure prediction. Nucleic Acids Res 33:W244–W248. doi:10.1093/nar/gki408. 15980461 PMC1160169

[12] Altschul SF, Gish W, Miller W, Myers EW, Lipman DJ. 1990. Basic local alignment search tool. J Mol Biol 215:403–410. doi:10.1016/S0022-2836(05)80360-2. 2231712

[13] Laslett D, Canback B. 2004. ARAGORN, a program to detect tRNA genes and tmRNA genes in nucleotide sequences. Nucleic Acids Res 32:11–16. doi:10.1093/nar/gkh152. 14704338 PMC373265

[14] Lowe TM, Chan PP. 2016. tRNAscan-SE On-line: integrating search and context for analysis of transfer RNA genes. Nucleic Acids Res 44:W54–W57. doi:10.1093/nar/gkw413. 27174935 PMC4987944

